# Co-circulation of influenza A(H1N1)pdm09 and influenza A(H3N2) viruses, World Health Organization (WHO) European Region, October 2018 to February 2019

**DOI:** 10.2807/1560-7917.ES.2019.24.9.1900125

**Published:** 2019-02-28

**Authors:** Hannah Segaloff, Angeliki Melidou, Cornelia Adlhoch, Dmitriy Pereyaslov, Emmanuel Robesyn, Pasi Penttinen, Sonja J Olsen, WHO European Region and the European Influenza Surveillance Network

**Affiliations:** 1 World Health Organization (WHO) Regional Office for Europe, Copenhagen, Denmark; 2 European Centre for Disease Prevention and Control (ECDC), Stockholm, Sweden; 3 The members of the network are listed at the end of the article

**Keywords:** influenza, epidemiology, Europe, surveillance

## Abstract

In the World Health Organization European Region, the 2018/19 influenza season started in week 49 2018, crossing 10% virus-positivity in sentinel surveillance specimens. At week 5 2019, activity remained elevated with positivity rates at 55%. Both A(H1N1)pdm09 and A(H3N2) viruses circulated widely and detection levels in primary care and hospital settings were similar to past seasons. Hospitalisation data may suggest an increased susceptibility to A(H1N1)pdm09 virus in older age groups.

The influenza season in 2018/19 in the World Health Organization European Region has been elevated for 9 weeks, with both influenza A viruses circulating, but the distribution of viruses differs greatly by country. Severity seems similar to past years that have had co-circulating influenza A viruses. Preliminary analyses of data from hospitalised influenza cases may suggest some increase in susceptibility to A(H1N1)pdm09 virus in older age groups.

## Influenza surveillance in the European Region

Every week, 50 of 53 Member States (MS) of the World Health Organization (WHO) European Region and Kosovo*report epidemiological and virological influenza surveillance data to the European Surveillance System (TESSy), hosted by the European Centre for Disease Prevention and Control (ECDC). Analyses of these data during the influenza season (weeks 20 to 40) are published jointly with the WHO Regional Office for Europe in a weekly influenza update, FluNewsEurope [1]. Influenza surveillance is primarily based on primary care sentinel sites collecting specimens from patients with acute respiratory infection (ARI) and/or influenza like illness (ILI) [2]. A subset of countries conducts surveillance of hospitalised influenza or respiratory infections. Here, we present data from week 40 (starting 1 October 2018) through week 5 (ending 3 February 2019). Data analysis of detected viruses, hospitalised cases and severe acute respiratory infection (SARI) includes cases reported to TESSy as at 11 February 2019; genetic and antigenic characterisation and antiviral resistance data are provided as at 7 February 2019.

## Start of the 2018/19 influenza season and activity as at 3 February

Influenza activity started to increase in early December (week 49 2018) and has continued into early February (week 5 2019); during these 9 weeks, more than 10% of the sentinel ARI/ILI specimens were positive for influenza (i.e. 10% threshold).

**Figure 1:**
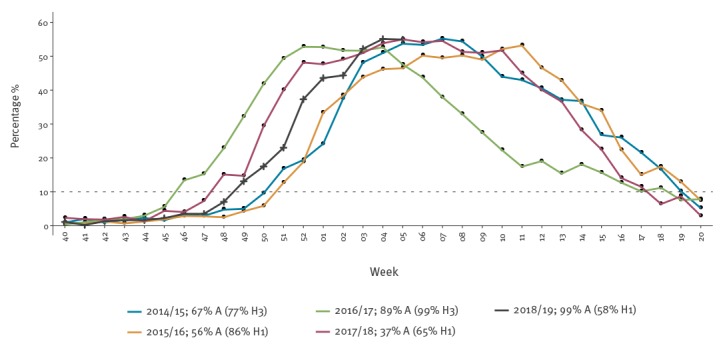
Percentage of sentinel ILI/ARI specimens testing positive for influenza, by season, 50 World Health Organization European Region Member States and Kosovo*, influenza seasons 2014/15–2018/19

ARI: acute respiratory infection; ILI: influenza-like illness.

In the 2018/19 season, the 10% threshold was crossed 1 week later than in the B-dominated 2017/18 influenza season, 3 weeks later than the A(H3N2)-dominated 2016/17 season and 2 weeks before the previous influenza A(H1N1)pdm09-dominated season of 2015/16. In addition, percent specimen positivity in week 5 2019 was 55%, comparable to what was observed during the past two seasons (Figure 1) [3,4].

Legend depicts seasons, percentage of sentinel detections that were influenza A and prevalence of most common influenza A subtype among all subtyped A viruses.

*This designation is without prejudice to positions on status, and is in line with United Nations Security Council Resolution 1244/99 and the International Court of Justice Opinion on the Kosovo Declaration of Independence.

The increase in influenza activity was first reported in the eastern part of the Region, with Georgia, Kyrgyzstan and Ukraine reporting medium influenza intensity by week 49 2018. These increases coincided with a steep rise in influenza virus detections from hospitalised SARI cases, with all three countries reporting over 50% percent positivity in week 50 2018. In Georgia and Kyrgyzstan detections were almost all influenza A(H1N1)pdm09, but in Ukraine A(H3N2).

Influenza activity has continued to intensify across the Region. In week 5 2019, the median positivity of sentinel specimens by MS was 57% with several countries, particularly in the southern and eastern part of the Region, reporting it as an intense influenza season. Turkey and Israel (countries where influenza A(H3N2) viruses circulated predominantly), reported weekly consultation rates for ILI exceeding those of the previous two seasons (2016/17 and 2017/18). Georgia and Albania, who had influenza A(H1N1)pdm09 viruses circulating predominantly, observed more SARI cases and higher percent positivity for influenza than in previous seasons. While in most countries reported activity was increasing or levelling off in week 5 2019, some countries in the southern and eastern European Region reported decreasing activity.

## Virology

### Influenza A

**Figure 2:**
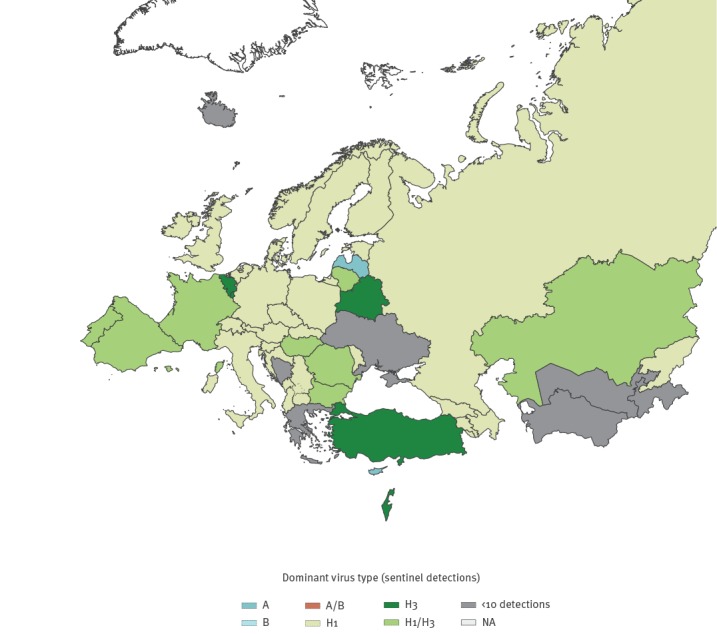
Distribution of dominant patterns of influenza virus type circulating, World Health Organization European Region, week 40 2018–week 5 2019

As at week 5 2019, 58% of all 7,437 subtyped influenza A viruses were influenza A(H1N1)pdm09 and 42% were influenza A(H3N2) among influenza cases detected though general practitioner sentinel systems; distribution of circulating influenza A subtypes varied by country (Figure 2). Influenza A(H1N1)pdm09 and A(H3N2) co-infections were reported and a seasonal reassortant influenza A(H1N2) virus was detected in Sweden [5].

Dominance of virus type/subtype is based on at least 60% of influenza virus detections from sentinel surveillance and co-dominance is based on between 40% and 60%. Calculations only considered valid if at least 10 viruses were detected from sentinel systems.

As at week 5 2019, all 767 antigenically characterised A(H1N1)pdm09 viruses were similar to the season’s vaccine virus, A/Michigan/45/2015. All of the 826 genetically characterised A(H1N1)pdm09 viruses belonged to subgroup 6B.1 represented by A/Michigan/45/2015. The majority (68%; n = 78) of antigenically characterised A(H3N2) viruses were similar to this season’s vaccine virus A/Singapore/INFIMH-16–0019/2016. Genetically, 81% (n = 432) belonged to clade 3C.2a and of these, the majority (83% n = 358) were in the 3C.2a1b group. Viruses belonging to clade 3C.3a were reported by some countries.

### Influenza B

Given the low prevalence, few influenza B viruses were genetically or antigenically characterised. All four B/Yamagata viruses antigenically characterised were similar to the quadrivalent vaccine virus and assigned to clade B/Phuket/3072/2013 of the quadrivalent vaccine virus. Three of four antigenically characterised B/Victoria viruses were similar to the trivalent influenza B vaccine virus. Among the 12 B/Victoria lineage viruses genetically characterised, 4 of 12 were assigned to the B/Colorado/06/2017 group containing the trivalent vaccine virus (HA1 double amino acid deletion, Δ162–163) and 5 of 12 were assigned to the B/Hong Kong/269/2017 group (HA1 triple amino acid deletion, Δ162–164). These triple deletion viruses are antigenically distinct from the vaccine virus and few have previously been detected.

### Neuraminidase inhibitor susceptibility

Neuraminidase inhibitor susceptibility was assessed for 897 viruses (A(H1N1)pdm09 = 602, A(H3N2) = 283; B = 12). Three A(H1N1)pdm09 viruses carried amino acid substitution H275Y in neuraminiase indicative of highly reduced inhibition by oseltamivir, one A(H3N2) virus showed evidence of reduced inhibition to oseltamivir and one B virus showed evidence of reduced inhibition to zanamivir in phenotypic tests.

## Severe acute respiratory infection (SARI) cases

**Figure 3:**
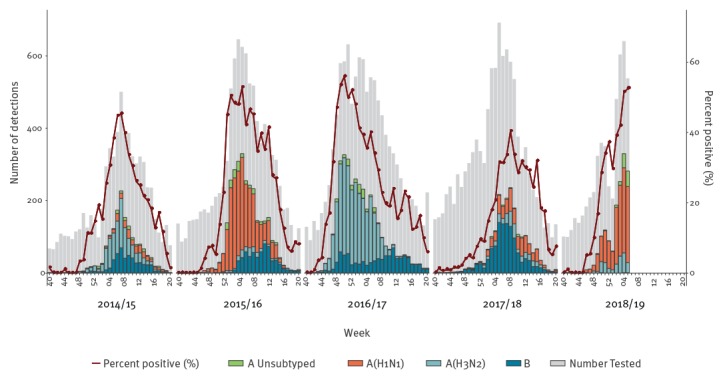
Number^a^, subtype and percent positivity of specimens from severe acute respiratory infection surveillance by season, World Health Organization European Region Member States, influenza season 2014/15–2018/19

SARI: severe acute respiratory infection; MS: Member State.

^a ^Number of countries conducting surveillance for severe acute respiratory infections in hospitalised patients: 2014/15 (n = 12), 2015/16 (n = 17), 2016/17 (n=16), 2017/18 (n=18) and 2018/19 (n = 17).

As at week 5 2019, 17 countries that conduct SARI surveillance reported 23,929 hospitalised cases; A(H1N1)pdm09 virus was detected in 77% (1165/1521) of influenza-positive cases. The total number of SARI detections and percentage of positivity were similar to peak detection and positivity seen in the 2015/16 A(H1N1)-dominated and 2016/17 A(H3N2)-dominated influenza seasons and both the number of cases detected and positivity rates exceeded those seen in 2014/15 or 2017/18 seasons (Figure 3).

Over the five seasons, data on SARI detections was reported by a subset of MS: Albania, Armenia, Azerbaijan, Georgia, Kyrgyzstan, Kazakhstan, Republic of Moldova, Serbia, Russian Federation and Ukraine for all seasons. For a subset of seasons, data on SARI detections was reported by: Bosnia and Herzegovina, Belgium, Belarus, Cyprus, Montenegro, Tajikistan, Turkmenistan, Uzbekistan and Kosovo*.

*This designation is without prejudice to positions on status, and is in line with United Nations Security Council Resolution 1244/99 and the International Court of Justice Opinion on the Kosovo Declaration of Independence.

## Laboratory-confirmed influenza in hospitalised patients admitted to the intensive care unit

**Figure 4:**
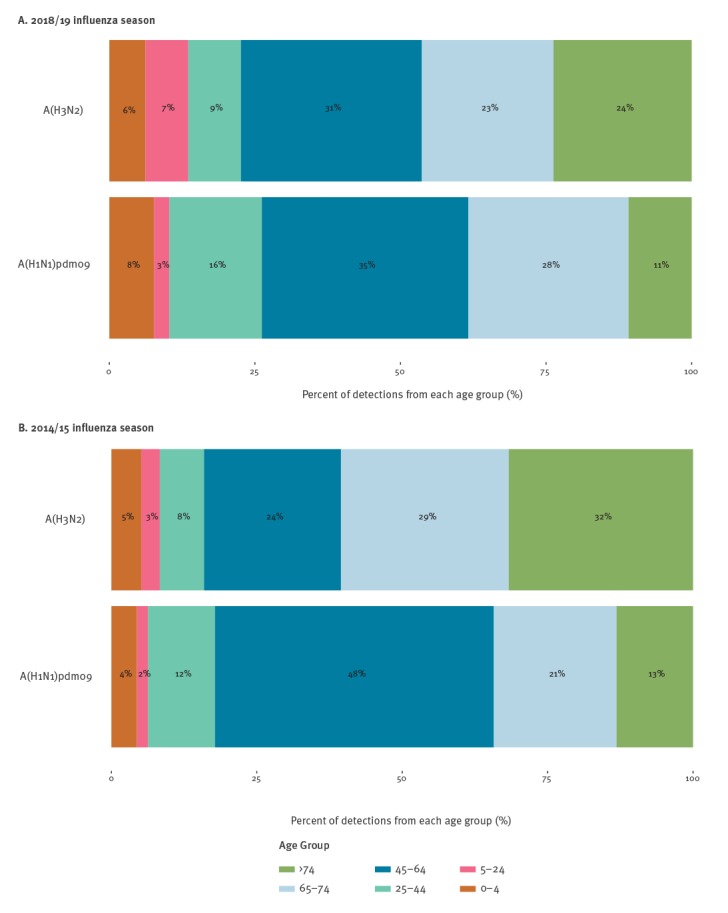
Age group distribution for influenza A(H1N1)pdm09 and A(H3N2) cases in the intensive care unit in the (A) 2018/19 and (B) 2014/15 influenza seasons, World Health Organization European Region Member States

As at week 5 2019, 12 countries that conduct case-based surveillance of influenza-positive hospitalisations reported 3,353 influenza-positive cases in persons admitted to the intensive care unit (ICU). Among influenza A viruses subtyped, 68% (378/555) were influenza A(H1N1)pdm09 and 32% (177/555) were influenza A(H3N2); 63% (956/1,511) of viruses were not subtyped. Compared with the 2014/15 season when both viruses also co-circulated, in 2018/19 the proportion of A(H1N1)pdm09 infected patients aged 24–44 years increased from 12 to 16% and that of patients aged 65–74 years from 21% to 27% (Figures 4). In contrast, the proportion of A(H3N2) infected patients aged 45–64 years (24–32%) increased.

Data on influenza-positive hospitalisations in the intensive care unit is collected by a subset of Member States; in 2014/15 data was reported by Denmark, Estonia, Finland, France, Ireland, Romania, Sweden and Slovakia. In 2018/19 the Czech Republic, Estonia, Russia and Ukraine also reported data and Slovakia did not provide data.

## Discussion and conclusions

Influenza activity in the WHO European Region has been high and increasing since early December (week 49 2018). As at 11 February 2019, the 2018/19 influenza season was dominated by influenza A viruses; both subtypes were co-circulating with a slight dominance of influenza A(H1N1)pdm09. Compared with past seasons dominated by influenza either A(H1N1)pdm09 or A(H3N2) (2014/15–2016/17), the current season has been similarly intense and SARI data suggest similar severity (although SARI data are only collected from a subset of countries in eastern Europe).

This is the tenth influenza season with circulation of A(H1N1)pdm09 virus since its initial appearance during the 2009 pandemic. Influenza A(H1N1)pdm09 seasons affect younger adults more severely; an estimated 65% of deaths from the first 12 months of the pandemic were in persons 18-64 [6]. In contrast, A(H3N2) affects adults 65 years and older more; about 80-90% of deaths from A(H3N2) occur in this age group [7,8]. As expected, among hospitalised patients in ICUs, we saw these age differences between influenza A(H1N1)pdm09 and influenza A(H3N2) cases. Persons aged 75 years and older accounted for a higher proportion of influenza A(H3N2) ICU admissions compared with A(H1N1)pdm09. However, when comparing ICU admissions from the 2014/15 season to the current season, we saw the relative proportion of adults aged 25–44 and 65–74 years increase among A(H1N1)pdm09-related ICU admissions and the proportion of those aged 45–64 years increase among cases with influenza A(H3N2). This changing age-distribution by subtype may, in part, reflect the ageing population of those with pre-pandemic exposure to the 1918-like A(H1N1) virus, who may have some residual immunologic protection [9]. This might explain the increased proportion of adults aged 65–74 years among A(H1N1)pdm09 detections in the current season, although this subset of data from 12 countries may not be generalisable and these early observations will require further, more rigorous, examination at the end of the season.

The relative proportion of circulating influenza A(H1N1)pdm09 and influenza A(H3N2) viruses varied by country and region this season. Recent interim vaccine effectiveness (VE) estimates indicate that the current vaccine is effective at preventing influenza virus infection, particularly by A(H1N1)pdm09 viruses, in children [10-12]. In recent years, the vaccine was less effective against influenza A(H3N2) viruses [13]. VE may vary across the Region based on the mix of circulating influenza subtypes and variation within the antigenic likeness of circulating A(H3N2) viruses with the egg-propagated A(H3N2) vaccine component. Despite its limitations, vaccination remains the most effective way to prevent influenza. Circulating influenza viruses during 2018/19 were largely susceptible to the antiviral drugs oseltamivir and zanamivir and these should be used as indicated by national guidelines.

## Note

* For the purposes of this publication, all references, including in the bibliography, to ‘Kosovo’ should be understood/read as ‘Kosovo (in accordance with Security Council resolution 1244 (1999))’. This designation is without prejudice to positions on status, and is in line with UNSCR 1244/1999 and the ICJ Opinion on the Kosovo declaration of independence.

## Members of the WHO European Region and European Influenza Surveillance Network

Albania: Iris Hasibra (Hatibi) and Artan Simaku

Armenia: Liana Torosyan and Shushan Sargsyan

Austria: Monika Redlberger-Fritz and Therese Popow-Kraupp

Azerbaijan: Oleg Salimov and Nazakat Abdullayeva

Belarus: Natallia Shmialiova and Natalia Gribkova

Belgium: Nathalie Bossuyt and Isabell Thomas

Bosnia and Herzegovina: Nina Rodic Vukmir and Diana Bastinac

Bulgaria: Neli Korsun and Anna Kurchatova

Croatia: Goranka Petrović and Vladimir Draženović

Cyprus: Maria Koliou and Despo Pieridou

Czech Republic: Martina Havlíčková and Jan Kynčl

Denmark: Ramona Trebbien and Tyra Grove Krause

France: Sibylle Bernard-Stoecklin and Sylvie van der Werf

Finland: Niina Ikonen and Outi Lyytikäinen

Georgia: Ann Machablishvili and Khatuna Zakhashvili

Germany: Silke Buda and Ralf Dürrwald

Greece: Athanasios Kossyvakis and Maria Exindari

Hungary: Zsuzsanna Molnár and Mónika Rózsa

Ireland: Linda Dunford and Niamh Murphy

Israel: Aharona Glatman-Freedman and Michal Mandelboim

Italy: Antonino Bella and Maria Rita Castrucci

Kazakhstan: Sultanov Meirim and Nussupbayeva Gaukhar

Kyrgyzstan: Otorbaeva Dinagul and Saparova Gulbarchyn

Latvia: Natalija Zamjatina and Raina Nikiforova

Lithuania: Asta Skrickienė and Algirdas Griškevičius

Luxembourg: Guillaume Fournier and Joel Mossong

Malta: Graziella Zahra and Jackie Maistre Melillo

Montenegro: Božidarka Rakočević and Zoran Vratnica

The Netherlands: Ron Fouchier and Adam Meijer

Norway: Olav Hugnes and Karoline Bragstad

Poland: Lidia B. Brydak and Katarzyna Luniewska

Portugal: Raquel Guiomar and Ana Paula Rodrigues

Republic of Moldova: Stefan Gheorghita and Alina Druc

Romania: Rodica Popescu and Odette Popovici

Russian Federation: Anna Sominina and Elena Burtseva

Serbia: Dragana Dimitrijevic and Svetlana Filipović-Vignjević

Slovenia: Katarina Prosenc and Maja Sočan

Spain: Amparo Larrauri and Francisco Pozo

Sweden: Mia Brytting and AnnaSara Carnahan

Switzerland: Ana Rita Gonçalves and Damir Perisa

Tajikistan: Tamanno Safarova and Niginamo Zakirova

The Republic of North Macedonia: Bosevska Golubinka and Kuzmanovska Maja

Turkey: Emine Avci and Ayse Basak Altas

Turkmenistan: Gurbangul Ovliyakulova and Ashirova Amansoltan

Ukraine: Iryna Demchishina and Oksana Artemchuk

United Kingdom: Jim McMenamin and Maria Zambon

Uzbekistan: Sultana Djemileva and Ravshan Rakhimov

WHO Collaborating Centre London: John McCauley and Rod Daniels

Radboud University Medical Center, The Netherlands: Tamara Meerhoff (WHO consultant)
